# What the future holds: the growing role of real-world evidence in health technology assessment in Malaysia

**DOI:** 10.1017/S0266462326103699

**Published:** 2026-04-30

**Authors:** Jing Sheng Lim, Kenneth Kwing Chin Lee

**Affiliations:** 1 School of Medicine and Health Sciences, Monash University Malaysia, Malaysia; 2 School of Pharmacy, Faculty of Health and Medical Sciences, Taylor’s University, Malaysia

**Keywords:** ASEAN, healthcare decision-making, health technology assessment, Malaysia real-world evidence

## Abstract

Health technology assessment (HTA) has long been the cornerstone of evidence-informed policy for healthcare decision-making. While randomized controlled trials (RCTs) remain fundamental, they do not always reflect the realities of everyday clinical practice. Real-world evidence (RWE) is increasingly recognized as an important complement, capable of capturing variations in population, setting, and practice that influence health outcomes. In Malaysia and across ASEAN, the growing focus on RWE reflects both global momentum and regional aspirations to strengthen data-driven policymaking. This perspective reflects on Malaysia’s experience in integrating RWE into HTA and considers how this evolution can inform regional efforts. It argues that the adoption of RWE signifies not only a methodological advance but a transformation in how evidence is conceptualized, governed, and applied. Developing robust data systems, analytical expertise, and cross-country collaboration will be essential to ensure that real-world data are translated into meaningful evidence for equitable and sustainable healthcare decisions.

Health technology assessment (HTA) provides a framework for evaluating the clinical, economic, and ethical aspects of healthcare interventions, supporting transparent, evidence-informed decisions about adoption, reimbursement, and prioritization (1;2). Traditionally, HTA has relied on randomized controlled trials (RCTs) to establish efficacy under controlled conditions. However, trial populations are often homogeneous, adherence differs in practice, and resource availability varies across healthcare settings (3).

The emergence of real-world evidence (RWE) addresses these limitations by offering insight into how technologies perform under routine conditions. RWE draws on data from sources such as electronic medical records, disease registries, claims databases, and patient-reported outcomes to provide a broader understanding of effectiveness and value (4). Globally, HTA and regulatory agencies now recognize RWE as a critical complement to RCTs. The shift is especially relevant in middle-income health systems that require context-sensitive, timely, and cost-efficient approaches to evidence generation (5).

Across ASEAN, interest in RWE is gaining traction as countries strive to align healthcare investment decisions with the needs and realities of their populations. Malaysia’s efforts to embed RWE within HTA processes illustrate this transition. Through the Ministry of Health and the Malaysian Health Technology Assessment Section, structured guidance on the generation and use of RWE has been introduced, marking an important institutional step toward integrating real-world data into policy decision-making (4).

This paper reflects on Malaysia’s evolving approach and its broader relevance in ASEAN, highlighting RWE not just a methodological refinement but as a shift toward a dynamic, learning-oriented HTA system.

## Relevance of RWE for HTA

HTA frameworks that rely primarily on RCT data face limitations when addressing policy questions requiring local context. RCTs measure efficacy under ideal conditions but often do not reflect the diversity of real-world populations, clinical workflows, or health system constraints. While these methodological limitations partly motivate RWE, its growing use is also driven by policy and institutional factors. These include earlier or conditional reimbursement of high-cost therapies, adoption of precision medicine and digital health technologies with limited population transferability, and adaptive reimbursement mechanisms requiring post-launch evidence (6). In these contexts, RWE complements clinical efficacy data by providing local insights on effectiveness, safety, resource use, affordability, scalability and long-term sustainability, supporting adaptive reimbursement, coverage reassessment, and budget impact analyses (7). These drivers highlight that RWE complements rather than substitutes for RCT evidence, offering actionable insights aligned with policy priorities and operational realities.

The relevance of RWE in Malaysia has become increasingly apparent through local and HTA-specific assessments. For example, analyses using hospital and registry data have demonstrated that applying Whole Exome Sequencing for children with suspected genetic disorders enabled earlier diagnosis and reduced unnecessary testing, leading to improved outcomes and cost savings (8). Similarly, evaluations of Continuous Glucose Monitoring Systems improved glycemic control in selected patients, though widespread use was not cost-effective at current prices (9).

RWE can also characterize disease burden, informing HTA and policy. For example, it has been used to quantify prevalence, healthcare utilization, and costs for major depressive disorder and colorectal surgical site infections, supporting prioritization and resource allocation (10;11).

Several Malaysian products have incorporated RWE to support regulatory and reimbursement decisions, illustrating its direct HTA relevance. Capmatinib for metastatic NSCLC with MET exon 14 skipping mutations, entrectinib for ROS1-positive NSCLC, palbociclib for male breast cancer, and post-marketing data for CoronaVac in children all leveraged RWE to contextualize clinical outcomes, treatment patterns, and resource use. These examples demonstrate how RWE complements RCT evidence to guide coverage, reimbursement, prioritization, and adaptive policymaking.

Taken together, these findings highlight that RWE is not simply supplementary but increasingly integral to informed HTA decision-making. As the volume and diversity of real-world data grow, traditional frameworks that place RCTs at the apex of evidence require re-evaluation. The key contributions of RWE to HTA and policy decision-making, alongside the requirements for credible evidence generation, are summarized in Table 1.

**Table 1.** Contributions of RWE to HTA and policy and associated requirements in Malaysia and ASEAN

RWE Contribution	Policy and HTA Implications	Capacity and Governance Consideration
Evidence on clinical effectiveness and safety	Supports coverage decisions, reimbursement, and post market evaluation	Skilled analysts, rigorous statistical methods, standardized and linked datasets
Resource utilization and costs	Informs budget impact analyses and economic evaluations	Access to claims or hospital costing data, EMR integration, data quality assurance
HRQoL and utility values	Enhances cost-utility analyses with contextually relevant patient-reported outcomes	Collection of local PROs, integration of PROMs into registries, standardized measurement protocols
Treatment patterns and access	Guides equitable allocation, coverage policies, and adherence monitoring	Cross-institutional data linkage, surveys for real-world adherence, routine utilization monitoring
Long-term outcomes	Facilitates continuous-learning HTA and evaluation of real-world trajectories	Longitudinal registries, standardized follow-up protocols, ethical oversight, and data governance

EMR, electronic medical records; HRQoL, health-related quality of life; HTA, health technology assessment; PROs, patient-reported outcomes; PROMs, patient-reported outcome measures; RWE, real-world evidence.

## From evidence hierarchy to evidence ecosystem

**Figure 1.**
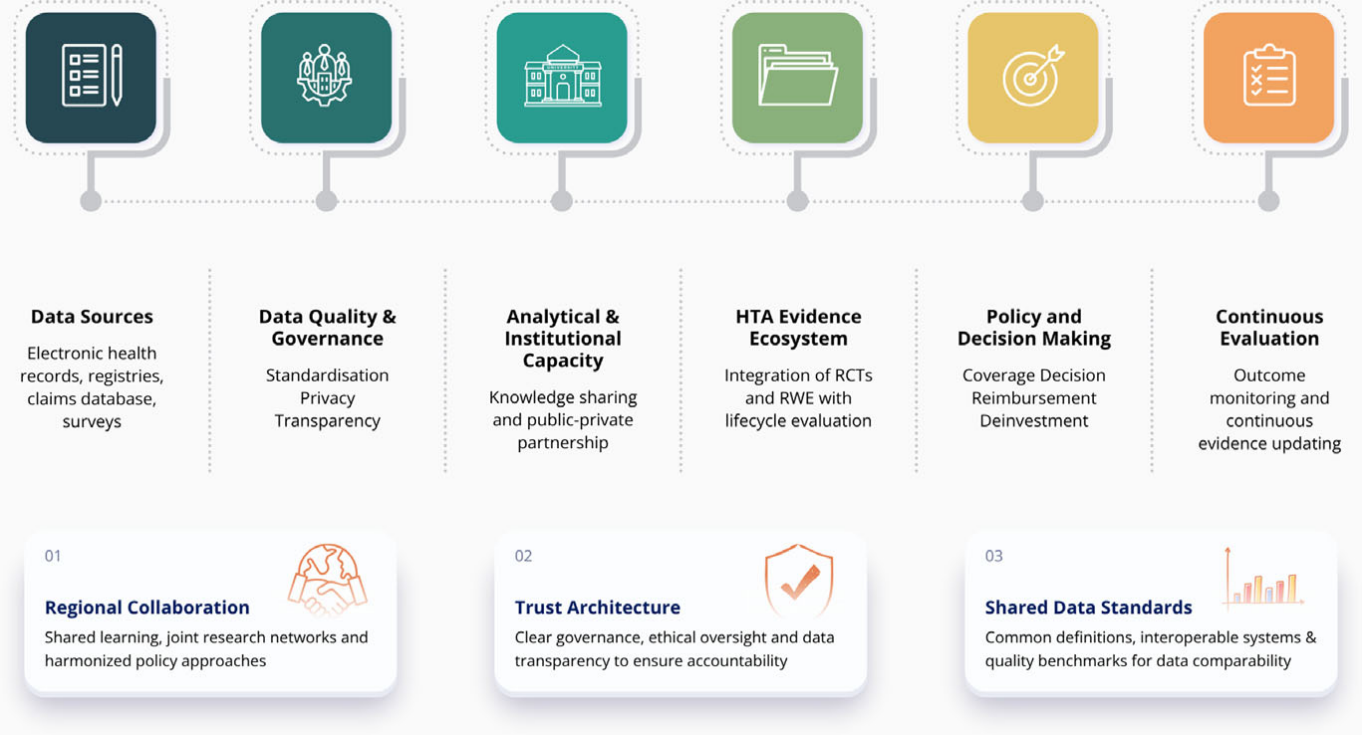
Conceptual framework of RWE flow from data generation through analysis and synthesis to application in health policy, with enabling factors.

Building on the increasing recognition of RWE’s policy relevance, the traditional evidence hierarchy which positions RCTs at the apex is being re-evaluated in favor of a more holistic evidence ecosystem, where diverse forms of data coexist and complement each other to support decision-making (12). Within this ecosystem, RCTs remain essential for establishing efficacy, while RWE deepens understanding of effectiveness, utilization, and cost within routine clinical practice. The conceptual framework in Figure 1 illustrates the dynamic flow of RWE from data generation through analysis and synthesis to its eventual application in health policy.

Malaysia’s evolving data environment provides a foundation for this approach. The country’s dual-tier healthcare system, comprising public and private providers, results in largely fragmented datasets. Although electronic health records (EHRs) are increasingly implemented in tertiary public hospitals, nationwide integration remains limited. Nevertheless, national registries and administrative claims databases, including the Casemix system and disease-specific registries, collect large volumes of routinely generated data that can be leveraged for HTA and policy-relevant analyses. For example, Casemix data have been used to assess utilization patterns and forecast costs for cardiovascular disease management in patients with and without diabetes, providing real-world insights into disease burden and resource allocation (13).

However, developing a functioning evidence ecosystem requires more than access to data. It demands coherent governance structures, methodological consistency, and a shared understanding of how different forms of evidence can complement one another. Building this ecosystem also involves shifting attitudes from viewing RWE as secondary to trial evidence, to recognizing its unique and indispensable contribution to policy relevance.

For ASEAN, the move toward evidence ecosystems represents a natural progression. Countries across the region share similar demographic and epidemiological transitions, as well as common constraints in research infrastructure. Collaborative efforts through networks such as HTAsiaLink can support shared methodological standards, regional data linkages, and mutual learning (14). A coordinated regional approach could accelerate the responsible use of RWE and foster greater policy convergence in the evaluation of health technologies.

## Methodological and governance considerations

Building on the discussion of why RWE matters for HTA and how evidence ecosystems can bring different data sources together, it is important to consider the practical factors that affect the credibility and usefulness of RWE. Beyond policy and institutional drivers, generating reliable RWE depends on addressing challenges around data quality, analytical capacity, and governance.

As aforementioned, data fragmentation remains a major barrier to RWE generation. In Malaysia, healthcare data are distributed across multiple systems and providers, often with inconsistent coding, incomplete fields, and limited interoperability. National registries, while valuable, cover only a subset of patient populations and some high-priority or rare conditions involve very small patient pools, making comprehensive analyses difficult. Similar issues are evident across ASEAN, where health information systems have developed unevenly and often lack standardization. Establishing data harmonization protocols and mechanisms for linkage across institutions would enhance the quality and usability of RWE throughout the region.

Analytical capability is another key determinant of success. Producing robust RWE requires a workforce proficient in advanced statistical techniques such as causal inference modeling, real-world cost-effectiveness analysis, and data linkage methods (15). Without this expertise, analyses risk bias and misinterpretation. Strengthening capacity through formal training programs and collaborative research initiatives would help build the analytical foundation needed for credible RWE integration.

Equally important are the governance and ethical frameworks that regulate data use. Privacy legislation in Malaysia, as in many ASEAN countries, provides necessary safeguards but can also constrain data sharing when interpreted conservatively (16). The challenge lies in balancing data protection with the societal value of secondary data use for public health research and policy. Transparent data governance structures, clear ethical guidelines, and strong institutional oversight are crucial for maintaining public trust while enabling legitimate research access.

A coordinated approach to these challenges could take the form of a regional governance model that establishes shared principles for data access, quality assurance, and ethics. Such a framework would support cross-country collaboration, enhance comparability of evidence, and strengthen the credibility of RWE-informed HTA throughout ASEAN.

## Opportunities and regional outlook

The increasing emphasis on RWE presents significant opportunities to enhance both national and regional capacity for evidence-informed decision-making. For Malaysia, integrating RWE into HTA represents an opportunity to align evidence generation with policy priorities and to strengthen the link between data and decision. When RWE is embedded within national health information systems, it not only supports HTA but also contributes to quality improvement, performance monitoring, and resource planning.

Across ASEAN, RWE provides a common platform for collaboration. The region’s shared epidemiological challenges and growing investment in digital health create a conducive environment for developing joint research infrastructure. Shared disease registries, regional RWE networks, and common methodological frameworks could substantially increase the volume and quality of locally relevant evidence. By pooling expertise and resources, ASEAN countries can reduce duplication, improve efficiency, and build collective credibility in international health policy discussions.

Public–private partnerships among government agencies, academia, and industry are also vital to this agenda. Each sector brings complementary strengths: policymakers provide direction and legitimacy, academic institutions contribute methodological expertise, and the private sector offers data and technical innovation. When managed transparently and ethically, these collaborations can enhance both evidence quality and policy relevance.

Finally, the use of RWE invites a redefinition of HTA’s role in health systems. Rather than being confined to pre-market evaluation, HTA can become a continuous-learning mechanism that monitors real-world outcomes and informs adaptive policy. This transformation would allow health systems to respond more quickly to changing technologies and emerging evidence, ensuring that resource allocation remains equitable and sustainable.

## Conclusion

The integration of RWE into HTA represents a paradigm shift in how ASEAN health systems generate and apply knowledge. In Malaysia, it signals progress toward a more adaptive and context-aware model of evaluation, while regionally it underscores the importance of collaboration and shared standards. Realizing RWE’s potential requires commitment to data quality, analytic capacity, and governance. Ultimately, the transformation lies not only in methods but in embedding continuous learning and evidence use within the complex realities of healthcare to support fair and effective policy decisions in mindset.
